# Angiotensin-Converting Enzyme (ACE) Inhibitor-Induced Angioedema Presenting After Long-Term Antihypertensive Therapy: A Case Report

**DOI:** 10.7759/cureus.100829

**Published:** 2026-01-05

**Authors:** Mohammed Khalid, Shifa Nafis

**Affiliations:** 1 Internal Medicine, Amina Hospital, Ajman, ARE; 2 Medicine, Ajman University, Ajman, ARE

**Keywords:** ace inhibitor-induced angioedema, ace inhibitors, airway management, angioedema, bradykinin-induced angioedema, delayed-onset angioedema, drug-induced angioedema, facial angioedema

## Abstract

Angioedema involving the airway is a serious medical condition that requires prompt recognition and treatment to prevent significant morbidity and mortality. Angioedema associated with angiotensin-converting enzyme (ACE) inhibitors can occur at any stage of therapy. While many patients develop symptoms early in treatment, others may remain asymptomatic for months or even years. Diagnosis is primarily clinical and relies on the presence of non-itchy, non-urticarial swelling in a patient receiving an ACE inhibitor, as there is no definitive laboratory test for ACE inhibitor-induced angioedema. ACE inhibitor-related angioedema is one of the most common types of non-histaminergic angioedema encountered in clinical practice. Here, we present a case of angioedema affecting the lips and tongue that developed years after ACE inhibitor initiation, resulting in difficult airway management and impending cardiac arrest. This case highlights the differential diagnosis of angioedema and the management strategies for a difficult airway.

## Introduction

Angioedema is a recognized adverse reaction to angiotensin-converting enzyme (ACE) inhibitors, occurring in a small proportion of users despite the widespread use of these medications for conditions such as hypertension, heart failure, and diabetic nephropathy [1]. While many reactions occur shortly after therapy begins, delayed onset is well documented, with some patients developing symptoms months or even years after starting treatment [2].

Swelling involving the tongue, lips, or upper airway can severely distort anatomy and complicate, or even prevent, intubation. Therefore, distinguishing ACE inhibitor-associated angioedema from hereditary or acquired C1 esterase inhibitor deficiency is crucial from both a clinical and management perspective. In drug-related cases, laboratory evaluation of complement C4 and C1 esterase inhibitor levels or functional activity is typically unremarkable despite the clinical presentation [3].

ACE inhibitor-induced angioedema is mediated by bradykinin accumulation rather than histamine release, which accounts for its delayed onset and poor response to conventional anti-allergic therapies [3]. This case is notable for its late presentation after years of stable therapy and the associated risk of acute airway compromise.

## Case presentation

A 56-year-old male presented to the emergency department with sudden-onset, progressive swelling of the lips and tongue. He initially experienced numbness in the tongue after dinner, followed by swelling that increased to the point that he was unable to swallow or eat and had difficulty breathing.

His medical history included type 2 diabetes mellitus and hypertension for the past five years. His medications were perindopril-indapamide (10/2.5 mg), sitagliptin-metformin (50/500 mg) twice daily, and bisoprolol 5 mg. He had been taking an ACE inhibitor for the past three years.

**Figure 1:**
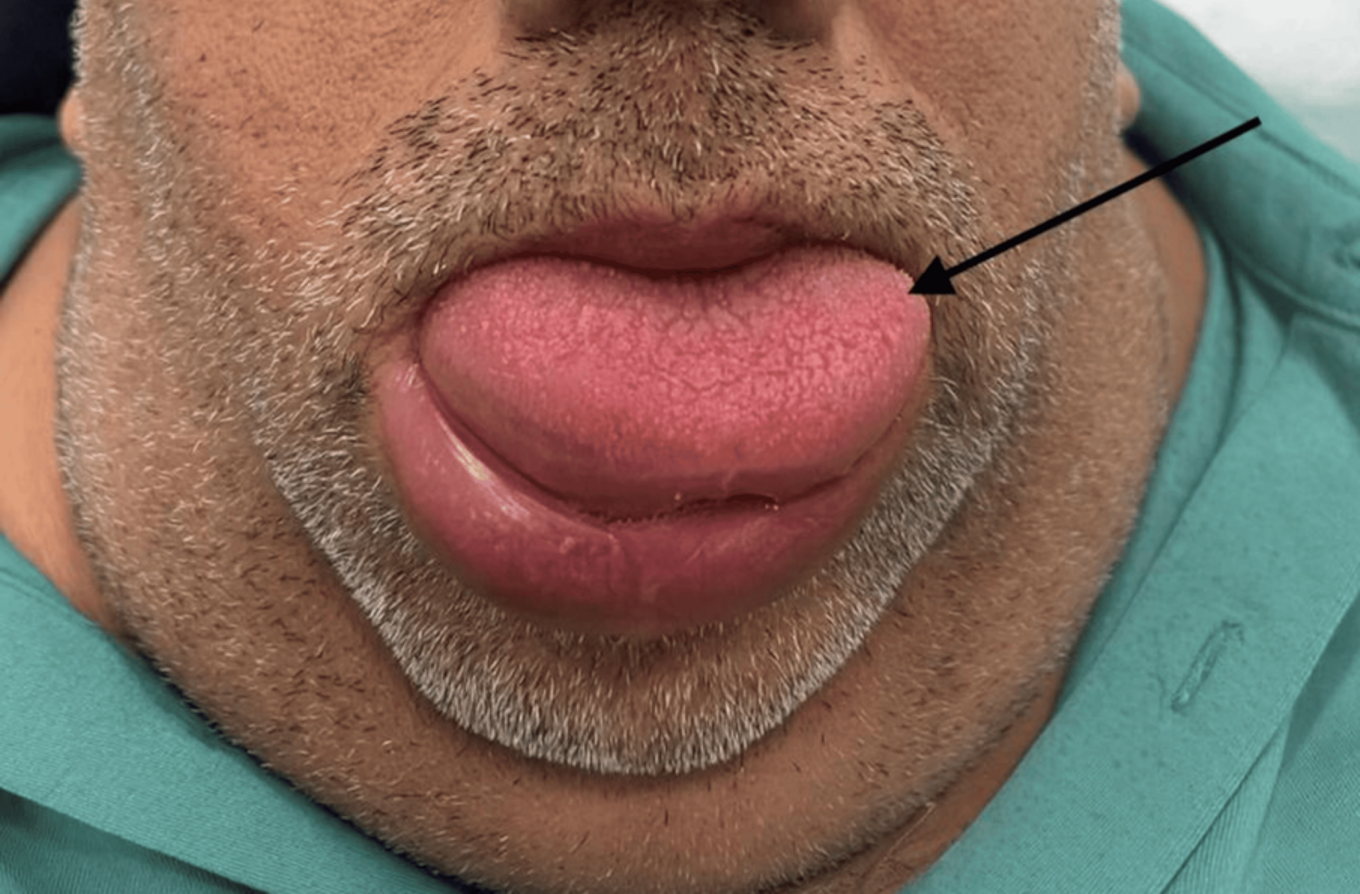
Patient presenting with acute swelling of the tongue and lips on admission Swelling of the tongue is pronounced, raising concern for potential airway compromise.

On admission, the patient was alert and oriented but unable to speak due to swelling of the tongue and lips (Figure 1). Vital signs were stable: temperature 37°C, pulse 80/min, respiratory rate 24/min, blood pressure 134/89 mmHg, and oxygen saturation 94% on room air. His body mass index was 43 kg/m² (height 167 cm, weight 120 kg), consistent with class III obesity.

Examination revealed a protruded tongue that could not be retracted, with severe swelling of the lips and tongue. The remainder of the systemic examination was unremarkable. Due to the pronounced tongue swelling and protrusion, there was concern for a difficult airway in the event of cardiac arrest. Video laryngoscopy or cricothyrotomy was considered if the patient’s condition deteriorated.

Differential diagnoses included hereditary angioneurotic edema versus ACE inhibitor-induced angioedema. The patient was admitted to the intensive care unit and placed on oxygen support.

Baseline laboratory investigations revealed leukocytosis, hyponatremia, and hyperglycemia (Table 1). These abnormalities were not directly related to ACE inhibitor-induced angioedema but likely reflected a mild stress response and underlying diabetes. Their presence did not affect the clinical diagnosis or management of angioedema.

**Table 1:** Baseline laboratory investigations obtained at admission

Parameter	Result	Reference range
Hemoglobin	14.5 g/dL	13.5-17.5 g/dL
White blood cell count	14 × 10³/µL	4-11 × 10³/µL
Platelets	219 × 10³/µL	150-450 × 10³/µL
Random blood sugar	192 mg/dL	<140 mg/dL
HbA1c	8%	<5.7% (normal); 5.7-6.4% (pre-diabetes); ≥6.5% (diabetes)
Sodium	133 mmol/L	135-145 mmol/L

The patient was treated with intravenous hydrocortisone, intravenous pantoprazole, intramuscular antihistamine, prednisolone, and oral cetirizine. Although ACE inhibitor-induced angioedema is bradykinin-mediated and typically unresponsive to these medications, they were administered in the emergency department as standard practice while the exact cause of angioedema was initially unknown. Most importantly, the ACE inhibitor was discontinued immediately. Blood glucose levels were monitored and managed with moderate doses of Humulin insulin, gliclazide, and metformin. On the following day, as his symptoms improved, he was transferred to the general floor to continue medical management.

**Figure 2:**
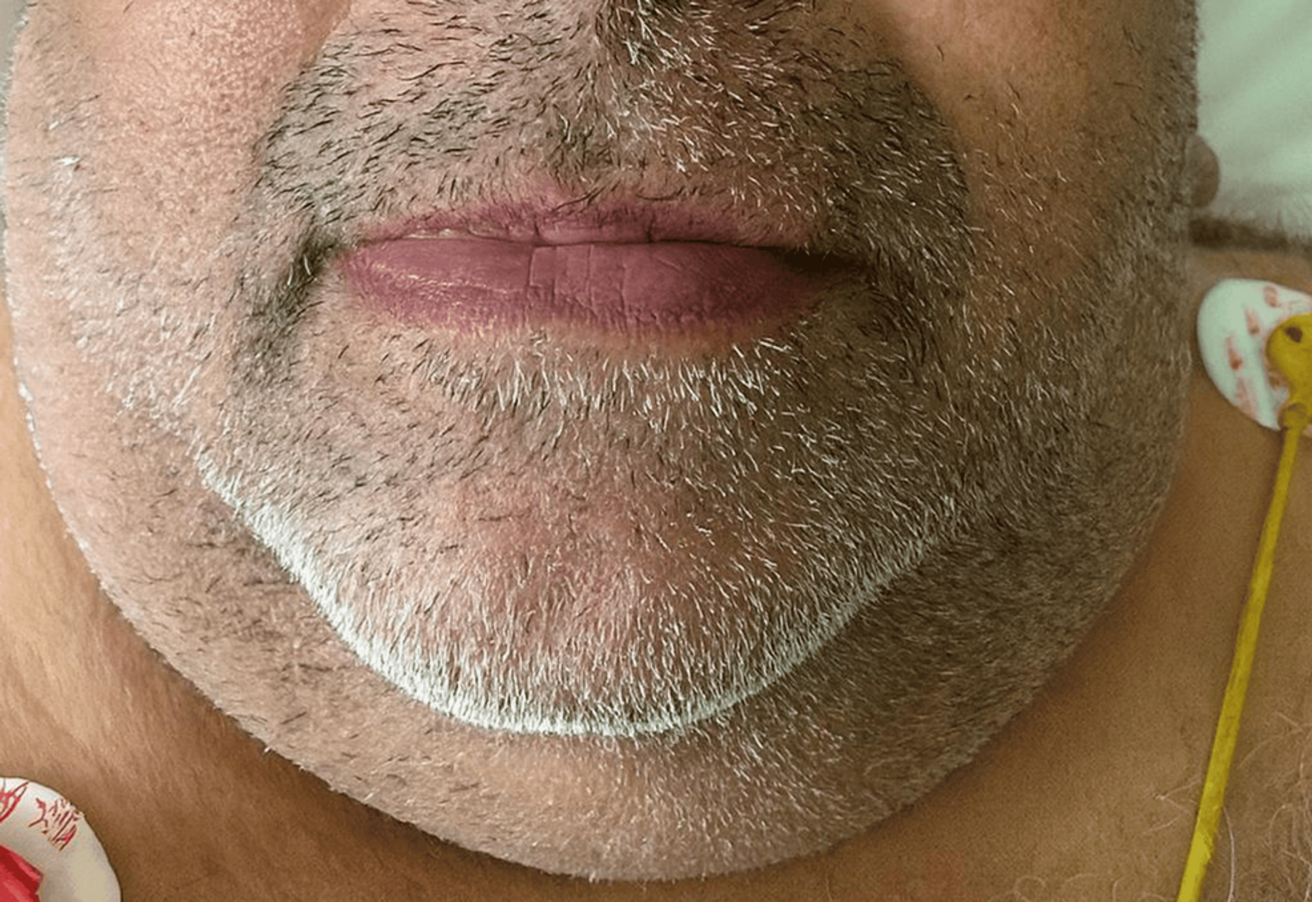
Posttreatment resolution of lip and tongue edema following discontinuation of the ACE inhibitor and supportive medical management ACE, angiotensin-converting enzyme

The tongue and laryngeal edema resolved (Figure 2). Further laboratory investigations were advised, including C1 esterase inhibitor levels, complement C4, and C1 esterase inhibitor functional activity. Once the patient’s condition stabilized, he was discharged home with instructions for outpatient follow-up. On follow-up, all additional laboratory tests were within normal limits (Table 2).

**Table 2:** Complement studies evaluating hereditary or acquired C1 esterase inhibitor deficiency

Parameter	Result	Reference range
Complement C4 level	34 mg/dL	20-50 mg/dL
C1 esterase inhibitor level	287 mg/L	210-390 mg/L
C1 esterase inhibitor activity	91%	70-130%

## Discussion

ACE inhibitor-induced angioedema results from increased bradykinin levels and can occur even after years of therapy without any previous reaction [3]. Unlike allergic or histamine-mediated swelling, this condition often does not present with urticaria or itching, which helps clinically differentiate the two conditions [4]. A single confirmatory diagnostic test is lacking; however, testing C4 levels and measuring C1 esterase inhibitor concentration and function is useful, as these studies help exclude hereditary and acquired C1 esterase inhibitor deficiencies, conditions that present similarly but are managed differently [3,5].

Traditional medications for allergic reactions, such as antihistamines, corticosteroids, and epinephrine, are typically ineffective because they do not act on the bradykinin pathway [6]. However, several treatments used for hereditary angioedema have been described in case series, including C1 esterase inhibitor concentrate, icatibant (a bradykinin B2 receptor antagonist), ecallantide (a selective and reversible plasma kallikrein inhibitor), and even fresh frozen plasma, though outcomes have been variable between reports [7].

Upper airway swelling requires immediate evaluation. Orotracheal intubation may be difficult or impossible due to the distortion of normal anatomy from the tongue and laryngeal edema. When feasible, advanced airway procedures such as awake fiberoptic intubation or video laryngoscopy should be used, with early involvement of experienced airway personnel. Because repeated unsuccessful intubation attempts can result in total airway obstruction and circulatory collapse, clinicians must be prepared for emergency surgical airway management [8].

## Conclusions

ACE inhibitor-induced angioedema remains a serious condition because it can develop suddenly after years of stable therapy and may rapidly compromise the airway. Early recognition, urgent airway assessment, and timely intervention are critical to prevent major complications. This case highlights the importance of preparedness for a potentially difficult airway and maintaining a high index of suspicion for medication-induced angioedema in patients presenting with swelling of the lips, tongue, or oropharynx.

## References

[1] Messerli FH, Nussberger J (2000). Vasopeptidase inhibition and angio-oedema. Lancet.

[2] Orr KK, Myers JR (2004). Intermittent visceral edema induced by long-term enalapril administration. Ann Pharmacother.

[3] Vasekar M, Craig TJ (2012). ACE inhibitor-induced angioedema. Curr Allergy Asthma Rep.

[4] Kyrmizakis DE, Papadakis CE, Liolios AD (2004). Angiotensin-converting enzyme inhibitors and angiotensin II receptor antagonists. Arch Otolaryngol Head Neck Surg.

[5] Morimoto T, Gandhi TK, Fiskio JM (2004). An evaluation of risk factors for adverse drug events associated with angiotensin-converting enzyme inhibitors. J Eval Clin Pract.

[6] Moellman JJ, Bernstein JA, Lindsell C (2014). A consensus parameter for the evaluation and management of angioedema in the emergency department. Acad Emerg Med.

[7] Maurer M, Magerl M, Ansotegui I (2018). The international WAO/EAACI guideline for the management of hereditary angioedema—the 2017 revision and update. Allergy.

[8] Farkas H (2010). Management of upper airway edema caused by hereditary angioedema. Allergy Asthma Clin Immunol.

